# Immunological detection of pyrazine-2-carboxylic acid for the detection of pyrazinamide resistance in *Mycobacterium tuberculosis*

**DOI:** 10.1371/journal.pone.0241600

**Published:** 2020-11-05

**Authors:** Edgar A. Florentini, Noelia Angulo, Robert H. Gilman, Roberto Alcántara, Elisa Roncal, Ricardo Antiparra, Emily Toscano, Katherine Vallejos, Danni Kirwan, Mirko Zimic, Patricia Sheen

**Affiliations:** 1 Laboratorio de Bioinformática y Biología Molecular, Laboratorios de Investigación y Desarrollo, Facultad de Ciencias y Filosofía, Universidad Peruana Cayetano Heredia, San Martín de Porras, Lima, Perú; 2 Department of International Health, Johns Hopkins Bloomberg School of Public Health, Baltimore, MD, United States of America; Consiglio Nazionale delle Ricerche, ITALY

## Abstract

Pyrazinamide (PZA) susceptibility testing in *Mycobacterium tuberculosis* (Mtb) is a current area of development and PZA-resistant strains are increasingly prevalent. Previous studies have demonstrated that the detection of pyrazinoic acid (POA), the metabolite produced by the deamidation of PZA, is a good predictor for PZA resistance since a resistant strain would not convert PZA into POA at a critical required rate, whereas a susceptible strain will do, expelling POA to the extracellular environment at a certain rate, and allowing for quantification of this accumulated analyte. In order to quantify POA, an indirect competitive ELISA (icELISA) test using hyperimmune polyclonal rabbit serum against POA was developed: for this purpose, pure POA was first covalently linked to the highly immunogenic Keyhole Limpet Hemocyanine, and inoculated in rabbits. A construct made of bovine serum albumin (BSA) linked to pure POA and fixed at the bottom of wells was used as a competitor against spiked samples and liquid Mtb culture supernatants. When spiked samples (commercial POA alone) were analyzed, the half maximal inhibitory concentration (IC50) was 1.16 mg/mL, the limit of detection 200 μg/mL and the assay was specific (it did not detect PZA, IC50 > 20 mg/mL). However, culture supernatants (7H9-OADC-PANTA medium) disrupted the competition and a proper icELISA curve was not obtainable. We consider that, although we have shown that it is feasible to induce antibodies against POA, matrix effects could damage its analytical usefulness; multiple, upcoming ways to solve this obstacle are suggested.

## Introduction

Tuberculosis (TB) is the leading infectious cause of morbidity and mortality worldwide. In 2017, approximately 1.3 million deaths and 10 million new cases were reported [1]. During this period 3.5% of new TB cases and 18% of previously treated cases were reported as having multi-drug resistance (MDR-TB) [1], with low and middle-income countries impacted the most [2].

Pyrazinamide (PZA) is a drug that has been used to treat TB since 1952 [3], and is presently included in both first- and second-line regimens [4]. Currently, the prevalence of PZA resistance is not well known. However, high rates of mortality have been reported when PZA is administered empirically to patients who are infected with PZA-resistant strains [5]. PZA resistance is associated with resistance to other TB medications: current estimates indicate that 50% of MDR-TB cases are also PZA resistant [6–8] and, alarmingly, the emergence of *Mycobacterium tuberculosis* (Mtb) strains resistant to all known anti-TB drugs has been reported [9]. This is likely to be caused by the mismanagement of pharmacological protocols. This scenario invokes the notion that testing for PZA resistance would be key to effective prescribing in TB, yet this is not routinely performed.

Despite its importance, the mechanism of action and resistance of PZA is not completely understood. Briefly, PZA is converted to POA by pyrazinamidase/nicotinamidase (PZAse) action in the mycobacterial cytoplasm. Once POA is produced it effluxes to the more acidic extracellular environment, where it is protonated. It then re-enters the cytoplasm where it reportedly acidifies the cytoplasm and interacts with different intracellular targets, resulting in cellular death [10–13].

Phenotypic Drug Susceptibility Testing (DST) for PZA is not included in routine testing for antimycobacterial drug resistance due to technical limitations specific to PZA. These limitations include a low critical concentration of PZA, and media alkalization by the inoculum, which is in conflict with the necessity to use a media pH close to neutrality [3, 14–17]. The way these tests should be ideally working is by indicating is by easily inform the analyst if the isolate is capable of surviving after exposure to PZA, in order to establish the optimal drug regimen to a particular patient. PZA *in vitro* testing however presents some intrinsic difficulties that have been delaying the standardization of its implementation and this has led to a lack of consensus [18].

The Wayne test and BACTEC MGIT 960 PZA (MGIT-PZA) are the phenotypic DSTs most commonly used to determine PZA susceptibility, although neither has been endorsed by the WHO. The Wayne test has a long turnaround time, and false-positive (resistance) reporting can occur due to subjective evaluation [15, 19]. On the other hand, MGIT-PZA has poor reproducibility, and a moderate to high rate of false resistance reporting due to a low critical concentration of PZA [6, 10]. Recently, a Microscopic Observation Drug Susceptibility (MODS) assay that incorporates the Wayne test (MODS-Wayne) has been described [20] and serves as a testing platform in the present study.

Analysis of the Mtb genome to detect drug resistance is now encouraged because of its ease of use and a short time to completion. In the case of PZA this approach seeks to identify mutations in the *pnc*A gene, which codifies for PZAse [5] However, the clinical utility of this method is reduced by the high variability of mutations along the *pncA* gene (no hot-spot is reported), the fact that not all mutations impair PZAse activity [7, 14, 21], and the lack of understanding of the relationship between each mutation and phenotypic resistance [6, 10]. Naturally-occurring Mtb strains that express PZAse with defective enzymatic activity have been found to be totally or partially resistant to PZA [13]. In addition, some *pncA* mutations are not associated with phenotypic resistance, and phenotypic resistance can also occur in the absence of any mutations, indicating that additional factors are also involved.

Previous studies [8, 12] have demonstrated that the detection of POA in Mtb culture medium is a good biomarker for PZA resistance: the absence of POA implies that no hydrolysis of PZA by PZAse is taking place and the isolate is, therefore, PZA resistant, whereas PZA susceptible strains produce POA under the same culture conditions. Measuring the concentration of the metabolite POA in the culture medium could thus be considered as a proxy indicator of resistance [12].

Immunological detection is based on the generation of antibodies against a selected analyte. Molecules must be of a certain size for this to occur. Those smaller than 1500 Daltons are not immunogenic [11], and therefore a common strategy is to conjugate the analyte with a larger carrier molecule, forming a hapten-carrier construct. This can then be administered to a mammal in order to elicit the induction of specific antibodies that can then be used in immunological assays.

Low molecular weight analytes such as POA are not structurally compatible with conventional sandwich assays [9]. In 1973 the competitive ELISA was first described [5], whereby competition between the analyte and a synthetic version of the same molecule enabled the detection of small analytes. In this study, we demonstrate that a competitive indirect ELISA assay can detect POA under controlled conditions.

## Materials and methods

### Chemicals, reagents and plasticware

The following reagents were purchased from Thermo Scientific: BSA (bovine serum albumin in PBS, Imject BSA # 77110), PMPI (p-maleimidophenyl isocyanate, “LINKER” in this study, #28100), Imject™ mcKLH (mariculture keyhole limpet hemocyanin, 5 x 20 mg, in PBS, #77600), and 1-Step™ Ultra TMB (3,3',5,5'-Tetramethylbenzidine, chromogenic ELISA substrate, # 34028). An anti-rabbit horseradish peroxidase-labeled goat IgG was acquired from KPL (#074–1506). Traut’s reagent (2-Iminothiolane HCl) was acquired from ProteoChem (Utah, US, #M3103). POA derivative (5-hydroxyl-2-pyrazinecarboxylic acid, “POA.OH”) was synthesized by Shanghai Haoyuan ChemExpress, Inc. Zeba™ Spin Desalting Columns, 7K MWCO, 2 mL (Thermo, #89889) were used for purification steps. A microplate with hydrophilic/hydrophobic surface (Maxisorp 96-Well Plates, Thermo Scientific, #439454) was employed throughout the study. Young adult rabbits (New Zealand, white females, 2.5–3.0 kg) were acquired from the National Institute of Health (Lima, Peru).

### Instruments and buffers

Plates were analyzed using the VersaMax microplate ELISA reader (Molecular Devices Co., Sunnyvale, CA, US). The following buffers were used: phosphate buffered saline (PBS, #21600010), composed of KCl 2.67 mM, KH2PO4 1.47 mM, NaCl 137.9 mM, Na2HPO4 8.1 mM; SEA BLOCK Blocking Buffer (steelhead salmon, i.e. *Oncorhynchus mykiss* serum in PBS, Thermo Scientific, #37527). Also, a “Traut’s solution” was prepared at a concentration of 54 μM in PBS buffer. Carbonate-bicarbonate coating buffer was acquired from Sigma (#C3041). A Nanodrop Spectrophotometer (Thermo Scientific #ND-2000) was used to assess the final concentration of constructs.

### Synthesis of constructs

**Fig 1:**
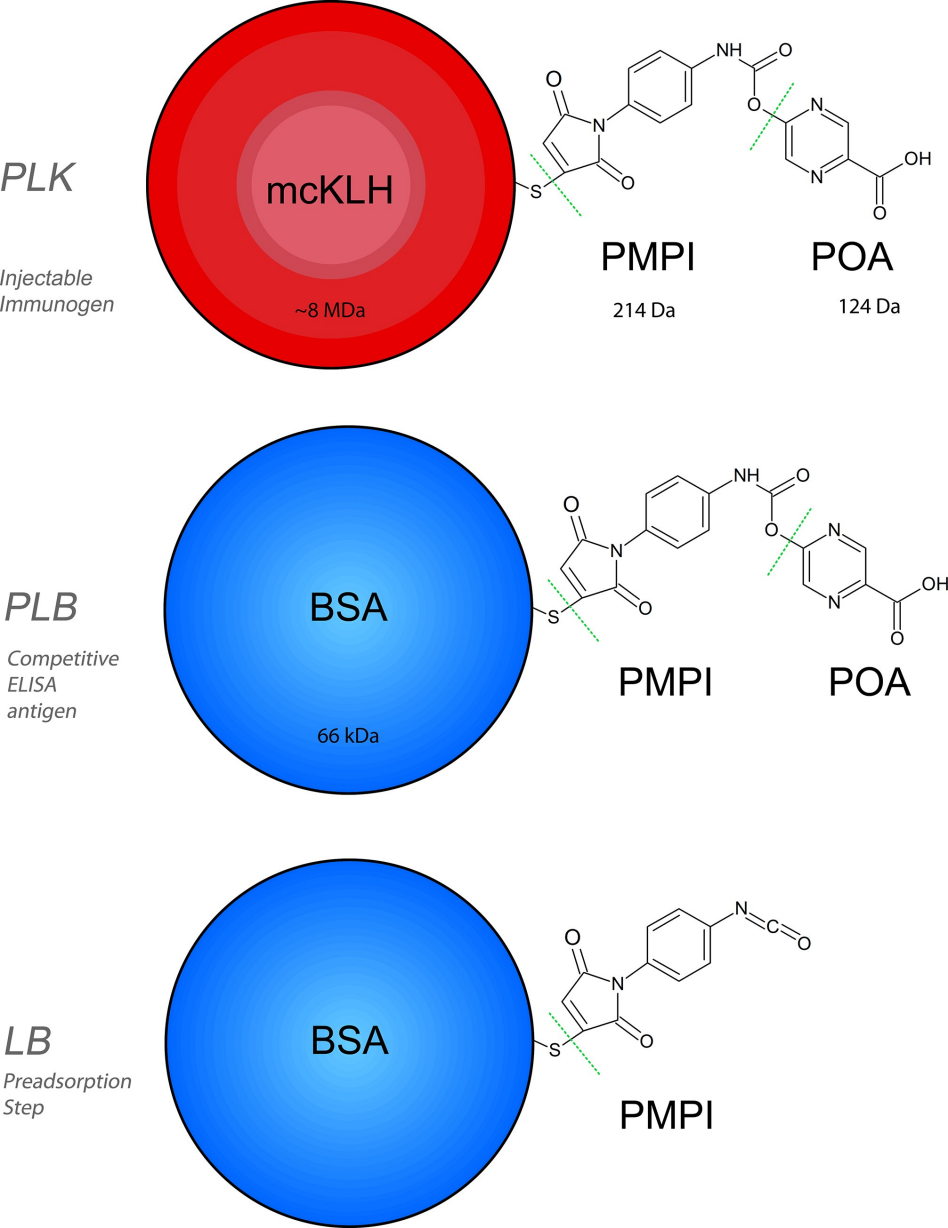
Structural formulas. Constructs (not to scale) used in the present study. Green dotted lines separate the original components. Acronyms are explained in Table 1. mcKLH being a massive molecule with an estimated weight of 8 million Daltons displays great immunogenicity in an mammal subject (rabbit). In comparison, molecular weight of the rest of reactant molecules is indicated.

All the chemical constructs synthesized in this study are listed in Table 1 and their structural formulas are depicted in Fig 1.

**Table 1:** Chemical constructs.

Denomination	Components^a^	Detailed structure	Estimated synthesis time	Role
PLK	POA.OH- LINKER-KLH	5-hydroxyl-2-pyrazinecarboxylic acid, p- maleimidophenyl isocyanate, 2-Iminothiolane, mcKLH	5h	Injectable immunogen
PLB	POA.OH- LINKER-BSA	5-hydroxyl-2-pyrazinecarboxylic acid, p- maleimidophenyl isocyanate, 2-Iminothiolane, BSA	5h	Competitive ELISA antigen
LB	LINKER BSA	p- maleimidophenyl isocyanate, 2-Iminothiolane, BSA	4h	Preadsorption step

^a^For this study, p-maleimidophenyl isocyanate was denominated as “LINKER”.

First, synthesis of the injectable immunogen POA.OH linker KLH (PLK) was carried out exploiting the specific reactivity of PMPI towards thiol groups. Briefly, 2 mg of carrier molecule mcKLH in 200 μL PBS buffer was incubated with 100 μL Traut’s solution (thiolation agent) for 90 minutes at room temperature, in constant rotation. Then, a 2 mL 7K MWCO Zeba column pre equilibrated with PBS was used to purify the precursor construct. This eluted volume was diluted to 630 μL in PBS. 70 μL organic solvent DMSO containing 600 μg linker agent PMPI and 1960 μg POA.OH, which was previously incubated away from light for 3 hours in constant rotation, were then added to the mix. This final mixture was then incubated for 2 hours and purified using a 2 mL 7K MWCO Zeba column, and stored at 4°C. The solution (approx. volume 700 μL) was mixed 1:1 with Freund's adjuvant and used for immunization. Next, synthesis of POA.OH linker BSA (PLB) -used as competitive ELISA antigen- was carried out in a similar way as the synthesis of PLK. BSA was employed due to avoid undesired anti KLH activity in the icELISAs. Briefly, 2 mg BSA in 200 μL PBS buffer was incubated with 100 μL Traut’s solution for 90 minutes at room temperature, in constant rotation followed by purification as with PLK (see above). Similar to PLK, 70 μL DMSO containing 600 μg PMPI and 1960 μg POA.OH was added to give a total volume of 700 μL. The PLB final mixture was incubated and purified as described for PLK. 20 μL aliquots of the final elution were stored in PCR microtubes at -80°C. Once an aliquot was thawed, it was stored at 4°C and was not re-frozen. Finally, the Linker-BSA (LB), used as a routine preadsorption step to discard antibodies against the PMPI linker, was synthesized in a similar fashion as PLB but omitting POA.OH (i.e. 70 μL DMSO containing only 600 μg PMPI). 20 μL aliquots of the final elution were stored in PCR microtubes at -80°C.

### Production of rabbit polyclonal hyperimmune antibodies against POA

Rabbits were inoculated with the PLK construct (see Table 1) following the schedule outlined in Table 2. Ethical approval for the employment of rabbits was obtained from the Institutional Ethics Committee of the Universidad Peruana Cayetano Heredia, with registration code 59921. PLK was thoroughly mixed with Freund’s adjuvant in a 1:1 ratio until a thick emulsion was formed, and then administered using a subcutaneous route in divided doses of approximately 100 μg per site to reach a total delivery of 1 mg per individual at each time point. Ear vein bleeding was performed using a Vacutainer system and a butterfly needle. For terminal bleeding, rabbits were anesthetized with ketamine (10 mg/100 g body weight) and xylazine (1 mg/100 g body weight), and whole blood volumes (30 mL) were obtained by cardiac puncture using a 21G needle. Whole blood was collected into red cap Vacutainer® (BD Plus Serum tube) tubes which were mixed 6 times by inversion, allowed to rest for 1h, later transported to laboratory facilities, and then centrifuged at 1300 x *g* for 10 min, all these steps took place at room temperature. Supernatants (i.e. serum) were aliquoted in 0.2 ml tubes (50 μL per aliquot) and stored at– 80°C. Hemolyzed samples were discarded.

**Table 2:** Immunization schedule.

Event	Day	Adjuvant	Amount of immunogen
Pre immunization serum collection	-10	-	-
PLK Priming Injection	0	Freund's Complete Adjuvant	1 mg
PLK Boosting Injection	14	Freund's Incomplete Adjuvant	1 mg
PLK Boosting Injection/Ear vein bleeding	28	Freund's Incomplete Adjuvant	1 mg
PLK Boosting Injection	56	Freund's Incomplete Adjuvant	1 mg
PLK Boosting Injection	84	Freund's Incomplete Adjuvant	1 mg
Terminal bleeding/Start of Anti IgG ELISA	98	-	-

Later, when icELISA analysis was performed, these serums underwent a routine preadsorption step in order to remove antibodies that were reactive against the linker moiety (PMPI) in the PLB. Serum was added to microwells pre-coated with LB and incubated overnight. Supernatants were collected and pooled, and this was used in all subsequent experiments.

### Indirect competitive ELISA (icELISA)

**Fig 2:**
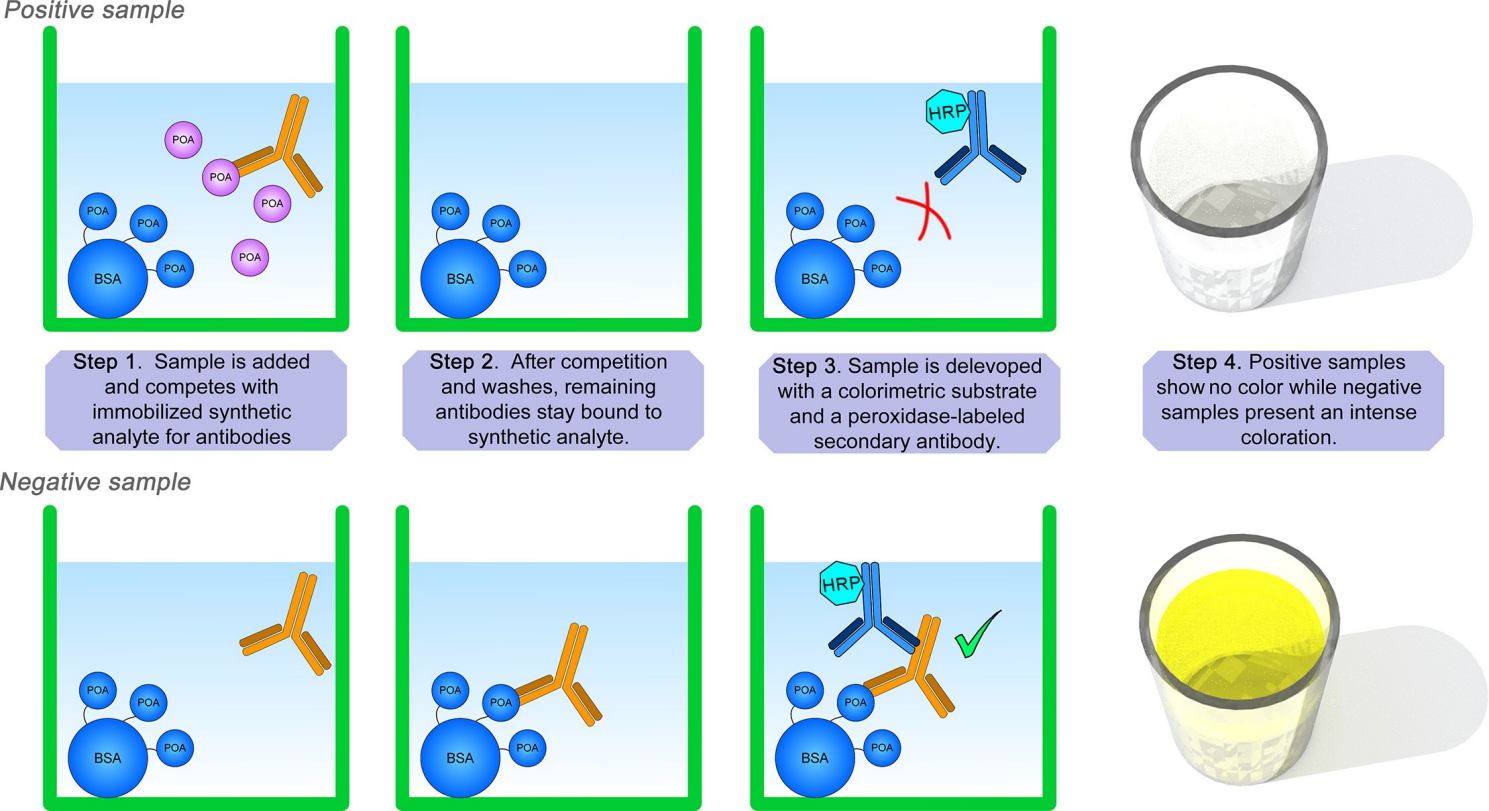
Indirect competitive ELISA. The principle of this assay can be explained as follows: In a positive sample, the POA analyte (purple) competes for rabbit polyclonal antibodies (orange) against synthetic analyte which is attached to BSA (blue, in this study denominated “PLB”). After several washes, a secondary anti-rabbit antibody (dark blue) labeled with horseradish peroxidase (HRP) is added to develop a colorimetric reaction that is inversely proportional to the amount of analyte: Positive samples will show absence of color and negative samples will show full intensity of color.

An indirect competitive ELISA (icELISA) (Fig 2) was performed, where PLB served as a competitive antigen against POA. Microwells were coated by overnight incubation at 4°C with PLB in 100 μL carbonate-bicarbonate buffer. The wells were washed, and PBS buffer spiked with different concentrations of POA (range 0.125 mg/mL– 4mg/mL) and final bleed (i.e. hyperimmune) antiserum were added. The plate was incubated for 1h at room temperature, then washed. A peroxidase-labelled secondary antibody was added, and the plate was incubated for 1h at room temperature. The colorimetric substrate was added then the plate was read using the VersaMax microplate ELISA reader (Molecular Devices Co., Sunnyvale, CA, US). Positive samples remained colorless, whereas negative samples underwent a color change (see Fig 2). POA concentrations were tested in duplicate, and a minimum of 20 assays were performed to confirm inter-assay consistency of the test (reproducibility). Antiserum, HRP, and PLB concentrations were standardized using a checkerboard titration.

The icELISA was performed using both pre-immunization and final bleed antiserum, and the ratio of reactivity was compared. The assay was also performed using PZA in place of POA, using the same range of concentrations, to test cross-reaction between the two compounds.

### Microscopic Observation Drug Susceptibility (MODS)-Wayne assay and icELISA of MODS-Wayne culture medium

The MODS-Wayne assay [15] was integrated into the workflow with the aim of detecting POA in the assay’s liquid culture medium. Ethical approval for the employed samples was obtained from the Institutional Ethics Committee of the Universidad Peruana Cayetano Heredia, with registration code 64196, which granted this study a Waiver of Ethical Approval Certificate. Briefly, sputum samples were decontaminated by mixing 1:1 with NaOH 2%, N-acetyl-L-cysteine 0.5%, and sodium citrate 1.45% in a 15 mL falcon tube, vortexed, and left for 15 min. 10 mL PBS was then added to neutralize the mixture, then samples were centrifuged at 3000 x *g*, 17°C for 15 min. The supernatant was discarded and the pellet resuspended in 6 mL 7H9-OADC-PANTA (a 45:5:1 mixture of 7H9 [3.1 mL glycerol, 1.25 g BD Biosciences Bacto™ casitone, and 5.9 g BD Difco Middlebrook 7H9 Broth in 900 mL of ultrapure water], BD OADC enrichment and BD PANTA™ antibiotic mixture, respectively). A sample aliquot of 100 μL was inoculated into two wells, one that was designated a control well, and the other designated the PZA well. 6 days after growth was observed in both wells (visible cord formation), PZA was added to the PZA well at a final concentration of 800 μg/mL, and both wells were incubated for 3 further days. Then, freshly prepared ferrous ammonium sulfate (FAS) was added to both wells at a final concentration of 1%. Visualization of a pink complex was taken to indicate the presence of POA, and therefore a PZA sensitive strain, whereas absence of a color change was taken to indicate PZA resistance. The plate was immersed in a water bath at 90°C for 30 minutes to sterilize the samples. 100 μL aliquots were then tested using the icELISA as described above. This was repeated 12 times using clinical samples positive for Mtb. Concordance between PZA resistance detected by the MODS-Wayne assay and by the icELISA was assessed.

In addition, wells containing 100 μL 7H9-OADC-PANTA were spiked with known concentrations of POA (range 0.125 mg/mL– 4mg/mL, as above). Ferrous ammonium sulfate (FAS) was added at a final concentration of 1%, and the icELISA was then performed. This was also repeated 12 times.

## Results

PLK, PLB, and LB were generated and purified as described. Quantification using UV-vis spectrophotometry determined concentrations of 1.8 mg/mL, 1.79 mg/mL and 1.91 mg/mL, respectively, which were all considered to fall within an acceptable range according to technical manuals from several commercial conjugation kits. Due to technical constraints, additional chemical verification of constructs through techniques such as thin layer chromatography was not employed, but since commercially mature kits were used, and being these synthesis procedures well-stablished, reasonably analytical success was expected.

These constructs were diluted in PBS and optimum dilutions that elicited the maximum absorbance in the absence of the analyte (Amax) using the minimum amount of analyte capable of inhibiting 50% of the signal (IC50) were found to be 1:400 for LB, 1:5,000 for PLB, 1:4,500 for HRP (working concentration of 222 ng/mL) and an antiserum dilution of 1:50,000.

When the icELISA was performed using pre- and post-immunization serum the ratio of reactivity was 1:49, indicating a significant immune response at the programmed time point (98 days post immunization).

**Fig 3:**
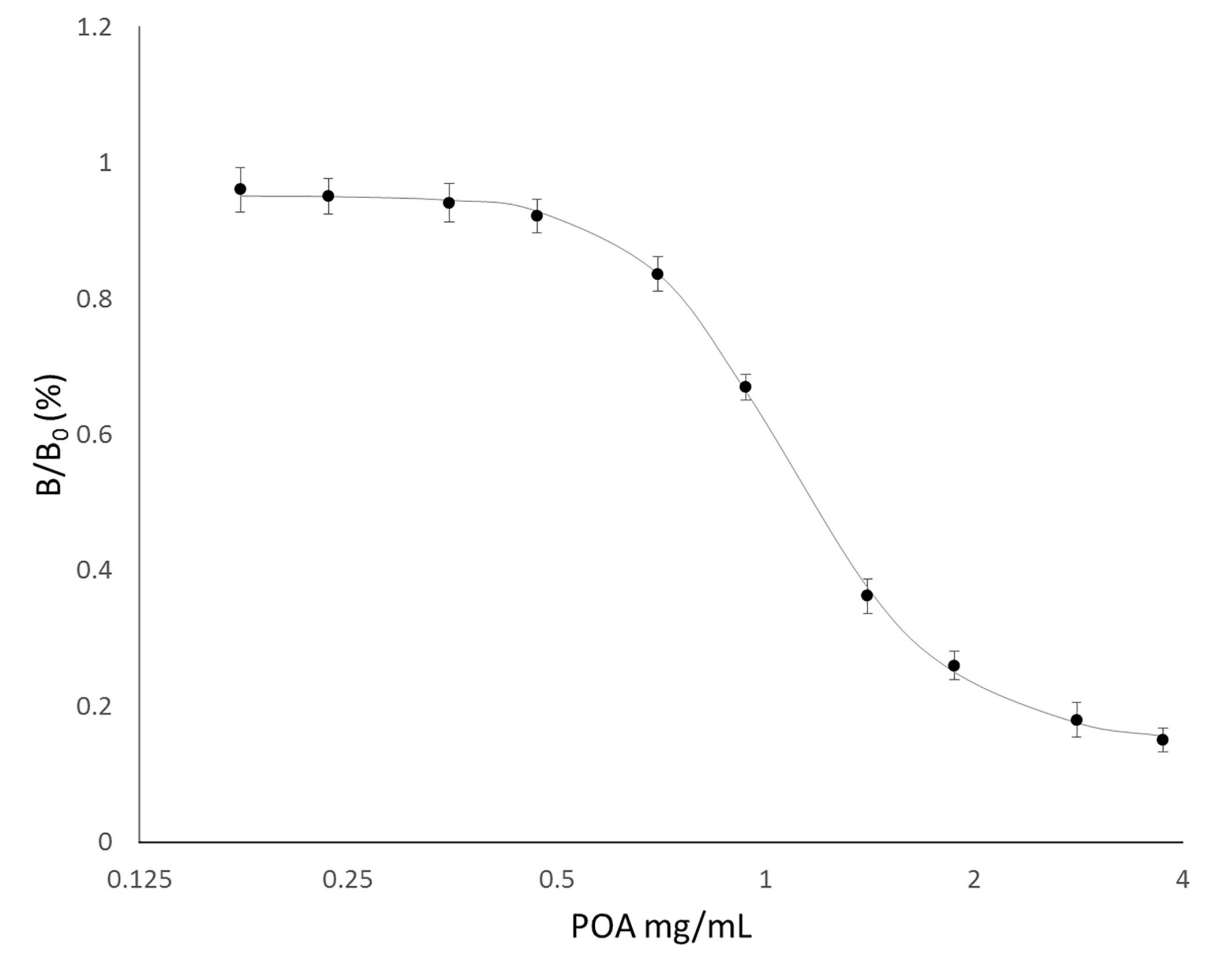
Optimized icELISA inhibition standard curve. The Y axis represents the ratio (%) of inhibition, inversely proportional to analyte concentration, where signal is maximal when no analyte is present or bound (B0) and reduced when increasing concentrations of analyte are bound to the BSA linked to POA (PLB) at the bottom of the well (B). Assays performed in triplicate. Data are presented as mean absorbances, and error bars represent SD. A 5 parameter logistic asymmetrical sigmoid curve was fitted with R2 = 0.9995. The X axis is displayed as log2 transformed.

**Fig 4:**
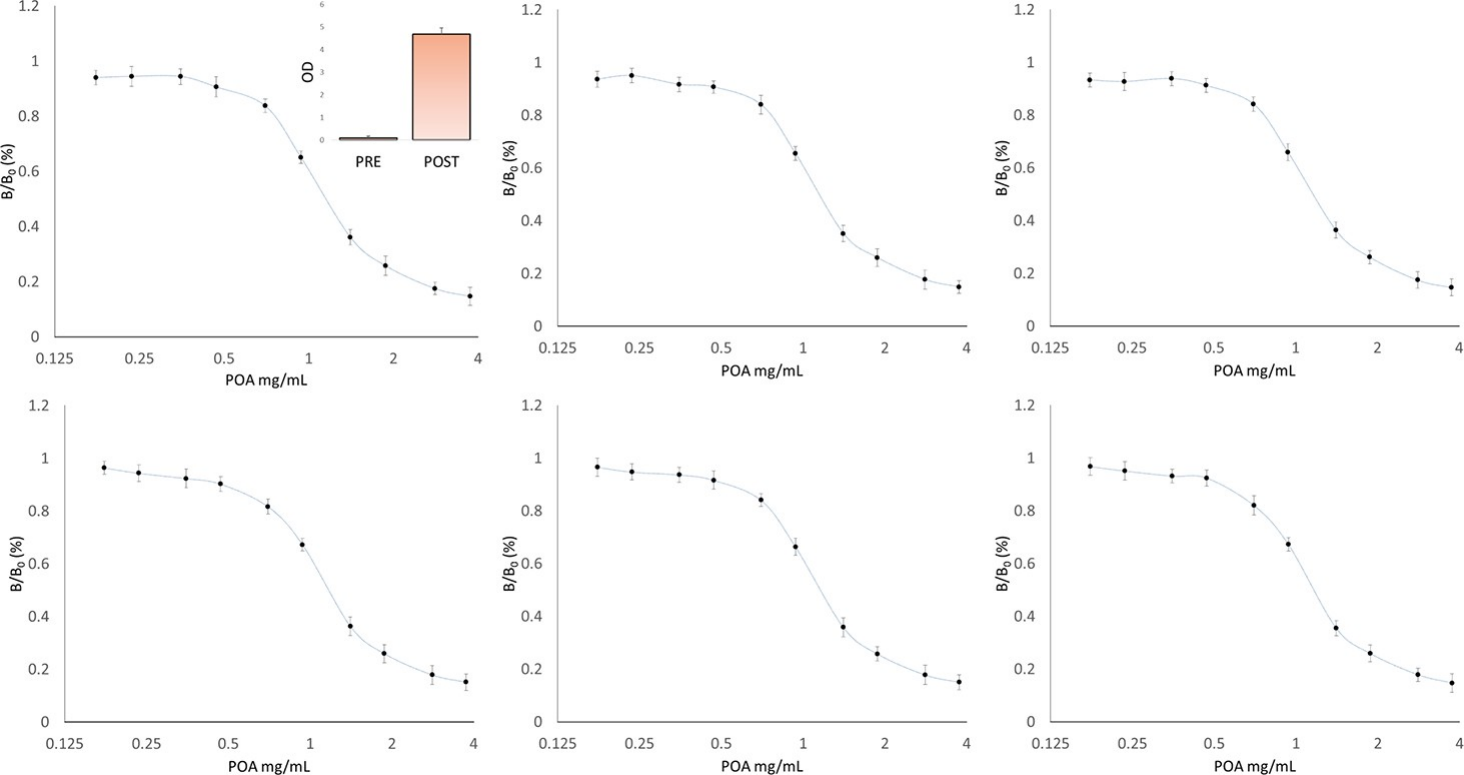
Reproducibility. Multiple inhibition curves showed inter-assay and inter-aliquot consistency when POA alone in liquid phase (no culture medium) was used. Inset: Representative bar chart showing that post immunization serum was highly reactive versus pre immunization against PLB, in a consistent manner, indicating a strong response (ratio = 1:49).

The parameters established during the optimization process described above generated the inhibition curve shown in Fig 3, with an IC50 of 1.16 mg/mL. The inhibition curve showed reproducibility across many repetitions on separate days (Fig 4), demonstrating stability of the polyclonal antibodies and a strong immune reaction elicited by the antigens.

When the icELISA was performed using PZA as the analyte instead of POA, no inhibition reaction was observed at all (IC50 >20mg/mL). This indicates that no cross-reactivity between PZA and POA occurred and also indicated a successful synthesis procedure. Of the 12 clinical samples tested, eight (66.7%) were positive according to the MODS-Wayne assay (Table 3). When these supernatants were tested using the icELISA the ODs obtained were erratic and no coherent regression curve was produced (not shown). A comparison between MODS-Wayne results and ELISA-POA was therefore not possible (Table 3).

**Table 3:** Tested samples.

Number	Sample Code	MODS-Wayne Result	ELISA-POA Result
1	TBN64	Positive	Indeterminate
2	TBN66	Positive	Indeterminate
3	TBN67	Positive	Indeterminate
4	TBN78	Positive	Indeterminate
5	TBN018	Positive	Indeterminate
6	TBN047	Negative	Indeterminate
7	TBN052	Negative	Indeterminate
8	TBN056	Negative	Indeterminate
9	TBN077	Negative	Indeterminate
10	TBN089	Positive	Indeterminate
11	TBN090	Positive	Indeterminate
12	TBN106	Positive	Indeterminate

A small cohort which included positive and negative MODS-Wayne samples was tested using the icELISA, with inderminate results.

**Fig 5:**
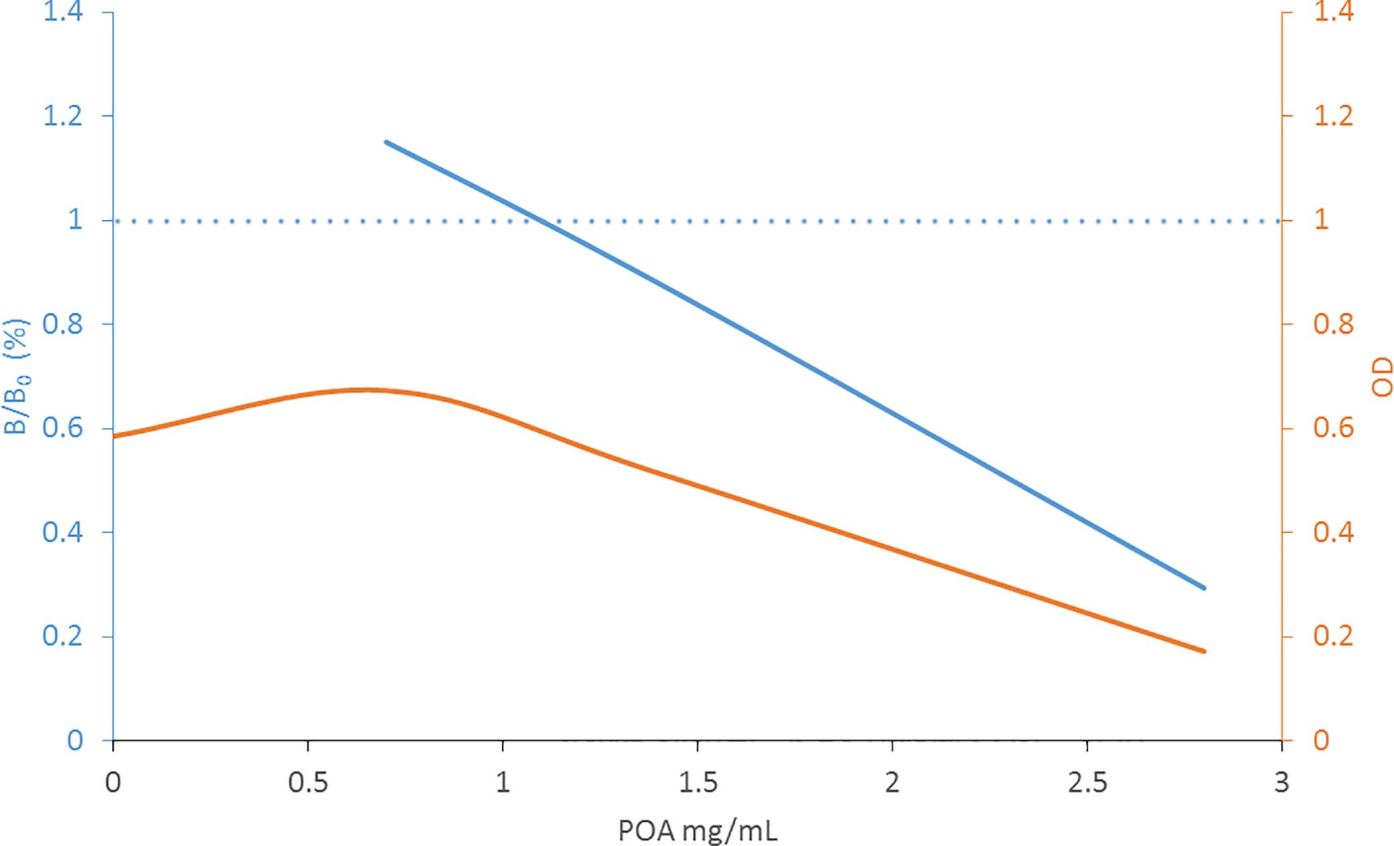
icELISA of POA in culture medium. 7H9-OADC-PANTA medium was used as reaction medium, where the orange line depicts raw ODs and the blue line represents B/B0 ratios (similar to Fig 4). The dotted blue line marks the B/B0 ratio of 1.

Similarly, when the icELISA was performed using 7H9 medium spiked with known concentrations of POA, instead of a sigmoidal inhibition curve a distorted, linear curve was observed. Fig 5 shows that the ratio between the sample with analyte bound versus sample with no analyte present (B/B_0_) surpassed 1 at low concentrations of POA, a technically impossible result, while crude ODs were significant (OD>0.1).

Following this finding, we sought to establish whether these distorted results were due to the presence of the culture medium or the FAS. We repeated the icELISA using known concentrations of POA in PBS and included FAS (0.1% or 1%). The inhibition curve generated was similar to the curve shown in Fig 3.

## Discussion

In this study, we have shown that it is possible to reliably induce polyclonal a hyperimmune antibodies in response to POA, a small molecule, in a mammalian model by haptenization. Similar findings have previously been achieved by other groups using small analytes such as proline (115 g/mol) [22]. We were then able to detect commercial POA in ultrapure water or PBS buffer using our icELISA, which consistently generated a specific competitive inhibition curve with an IC50 of 1.16 mg/mL (9.35 mM POA).

When designing an icELISA, critical aspects of immunogen efficiency to consider include establishing the correct exposure of antigenic determinants to ensure specificity and the length of the spacer arm: an arm that is too short may become masked by the carrier surface, whereas a long spacer arm could create heterogeneous tertiary structures, diluting its immunogenic potential [23]. In the present study, the POA derivative 5-hydroxyl-2-pyrazinecarboxylic acid was the only one that was commercially available, which was a starting constraint for the study. For that reason, only conjugation strategies capable of using the hydroxyl group were considered, and PMPI was chosen as the linker. This allowed us to expose the carboxyl group that differentiates it from PZA and enabled us to generate a specific immunoassay. Nevertheless, despite these limitations, the IC50 achieved by our assay was high (1.16 mg/mL) compared to other competitive immunoassay studies using small molecules such as aflatoxin g1 (278.321 g/mol, IC50: 17.18 ng/mL) [24].

The Wayne assay has been estimated as having a sensitivity of 0.5 mM POA, and the quantitative version as having a sensitivity of 0.05 mM [8], one and two orders of magnitude greater than the platform described in the present study, respectively. It is possible that the icELISA detection limit could be lowered by carrying out some additional optimization. Pioneering studies using *in vivo* models to generate antibodies typically used large numbers of immunized animals, which enabled the testing of a range of different routes of administration, immunization schedules, and adjuvants. This allowed for largescale screening of hyperimmunized sera [25], maximizing the probability of finding an individual or individuals with excellent response. This was not the case for our study, which was limited by our staff availability, facilities, and access to reagents. This method of trial and error could in future studies surpass this limitation and arrive at an optimal protocol for the generation in rabbits of polyclonal antibodies with very high affinity, and with an IC50 in the range of nanograms per milliliter.

The icELISA we have developed does not seem to tolerate the presence of MODS culture medium. The MODS liquid culture medium contains 7H9-OADC-PANTA, which comprises 21 soluble ingredients; in a competitive system, this creates a risk of interference. It may be possible to mitigate this by diluting or pre-purifying the samples [26]. This may be further compounded by the fact that POA is one of the smallest haptenized immunogens so far reported in the literature, to the best of our knowledge; it can be generalized that the smaller the hapten, the less diverse would be the useful polyclonal repertoire.

Overcoming the challenges in incorporating MODS culture medium into the icELISA assay would represent a significant step in the development of this assay and move towards a clinically useful test for the detection of PZA resistance in clinical strains. The MODS-Wayne assay is based upon attaining growth of Mtb in liquid culture medium (mean time to positivity 7 days) and, once growth is visible, the addition of PZA. Currently, the MODS-Wayne assay requires a further three days of bacterial growth before FAS can be added and the strain’s sensitivity to PZA revealed via the presence or absence of a color change. The icELISA would replace this final step, and thereby shorten the process by two days. This could be clinically important to patients who are being treated with an ineffective drug regimen.

The present study demonstrates that detection of POA using a competitive ELISA is possible, and serves as a primer to identify technical challenges in assay design and elucidate which novel technologies could be applied to solve them. Further work to optimize the assay is needed before a commercial translatable system can be developed. Routine microbiological tests should not be cumbersome, too expensive, nor impractical. Emerging ultra-sensitive applications such as immuno-PCR could integrate very well into our workflow and surpass all mentioned obstacles so far [27]; or, improved final step amplified detection strategies such as a biotin streptavidin system could be incorporated [28]. Upcoming cost/benefit analyses will give us more insight in this regard.

## Supporting information

S1 Table Curve readings. ODs conforming POA curve.
(XLSX)

S2 Table Antibody titration. ODs obtained in the titration of the antibody when PLB was added to the bottom of the well.
(XLSX)
