# Correction: Immunological detection of pyrazine-2-carboxylic acid for the detection of pyrazinamide resistance in *Mycobacterium tuberculosis*

**DOI:** 10.1371/journal.pone.0259439

**Published:** 2021-10-27

**Authors:** Edgar A. Florentini, Noelia Angulo, Robert H. Gilman, Roberto Alcántara, Elisa Roncal, Ricardo Antiparra, Emily Toscano, Katherine Vallejos, Daniela E. Kirwan, Mirko Zimic, Patricia Sheen

The ninth author’s name is incorrect. The correct name is: Daniela E. Kirwan.

The ORCID iD is missing for the ninth author. Author Daniela E. Kirwan’s ORCID iD is: 0000-0003-3989-0795.

The affiliation for the ninth author is incorrect. The correct affiliation is not indicated. Daniela E. Kirwan is not affiliated with #1 but with: Institute for Infection & Immunity, St George’s University of London, London, United Kingdom.

The following information is missing from the Funding statement: DEK is supported by Medical Research Council, UK Fellowship MR/P019978/2.
